# Correction of Biogeochemical-Argo Radiometry for Sensor Temperature-Dependence and Drift: Protocols for a Delayed-Mode Quality Control

**DOI:** 10.3390/s21186217

**Published:** 2021-09-16

**Authors:** Quentin Jutard, Emanuele Organelli, Nathan Briggs, Xiaogang Xing, Catherine Schmechtig, Emmanuel Boss, Antoine Poteau, Edouard Leymarie, Marin Cornec, Fabrizio D’Ortenzio, Hervé Claustre

**Affiliations:** 1 CNRS & Sorbonne Université, OSU Ecce Terra, 4 Place Jussieu, CEDEX 05, 75252 Paris, France; quentin.jutard@sorbonne-universite.fr (Q.J.); catherine.schmechtig@imev-mer.fr (C.S.); 2 National Research Council (CNR), Institute of Marine Sciences (ISMAR), Via del Fosso del Cavaliere 100, 00133 Rome, Italy; 3 National Oceanography Centre, Southampton SO14 3ZH, UK; nathan.briggs@noc.ac.uk; 4 State Key Laboratory of Satellite Ocean Environment Dynamics, Second Institute of Oceanography, Ministry of Natural Resources, 36 Baochubei Road, Hangzhou 310012, China; xing@sio.org.cn; 5 School of Marine Sciences, University of Maine, Orono, ME 04469, USA; emmanuel.boss@maine.edu; 6 CNRS & Sorbonne Université, Laboratoire d’Océanographie de Villefranche, 06230 Villefranche sur mer, France; antoine.poteau@imev-mer.fr (A.P.); edouard.leymarie@imev-mer.fr (E.L.); marin.cornec@imev-mer.fr (M.C.); fabrizio.dortenzio@imev-mer.fr (F.D.); herve.claustre@imev-mer.fr (H.C.)

**Keywords:** BGC-Argo, radiometry, quality control

## Abstract

Measuring the underwater light field is a key mission of the international Biogeochemical-Argo program. Since 2012, 0–250 dbar profiles of downwelling irradiance at 380, 412 and 490 nm besides photosynthetically available radiation (PAR) have been acquired across the globe every 1 to 10 days. The resulting unprecedented amount of radiometric data has been previously quality-controlled for real-time distribution and ocean optics applications, yet some issues affecting the accuracy of measurements at depth have been identified such as changes in sensor dark responsiveness to ambient temperature, with time and according to the material used to build the instrument components. Here, we propose a quality-control procedure to solve these sensor issues to make Argo radiometry data available for delayed-mode distribution, with associated error estimation. The presented protocol requires the acquisition of ancillary radiometric measurements at the 1000 dbar parking depth and night-time profiles. A test on >10,000 profiles from across the world revealed a quality-control success rate >90% for each band. The procedure shows similar performance in re-qualifying low radiometry values across diverse oceanic regions. We finally recommend, for future deployments, acquiring daily 1000 dbar measurements and one night profile per year, preferably during moonless nights and when the temperature range between the surface and 1000 dbar is the largest.

## 1. Introduction

The international Biogeochemical-Argo (i.e., BGC-Argo) program has revolutionized the way we acquire measurements of biogeochemically relevant variables in the open ocean [1,2]. In 2016, the Biogeochemical-Argo planning group has defined six core variables to accomplish the scientific and observational objectives of the program that include the study of the ocean carbon uptake and acidification, oxygen minimum zones and nitrate cycling, biological carbon pump, phytoplankton communities, and joint use with ocean color satellite observations [3]. In particular, to study phytoplankton dynamics and combine in-situ with remote sensing observations, radiometry, i.e., measurements of downwelling irradiance (E_d_) and photosynthetically available radiation (PAR), has been selected as a core variable.

Currently, the BGC-Argo program has accumulated more than 40,000 profiles of downwelling irradiance (between 0 and 250 dbar), acquired by more than 100 floats in the global ocean, across a variety of trophic and environmental conditions, and in remote regions (https://biogeochemical-argo.org/, accessed on 13 September 2021). These profiles have proved to be fruitful measurements for diverse applications. Downwelling irradiances at various wavelengths have been implied in the analysis of the bio-optical behavior of the global ocean [4] and the dynamics of dissolved organic matter [5,6], and for the validation of space-based ocean color measurements and products [7,8,9,10,11,12,13]. Besides, E_d_ and PAR have been widely used to understand particulate organic carbon fluxes and export [14,15,16], to study phytoplankton dynamics [17,18,19,20,21,22,23,24,25], and to improve numerical and radiative-transfer models [26,27]. 

Despite the relevant scientific results, some inconsistencies in deep radiometric measurements, where the lowest irradiances are expected, have been observed [8,9,28]. With time and through the analysis of acquired data, our knowledge on the sensor behavior has progressively improved and identified two main radiometer characteristics which are independent one from the other, neglected since the launch of the fleet in 2012. First, the dark measurements of the sensors are sensitive to the ambient temperature which ultimately reduces measurement accuracy, especially in the deep part of the profile where the remaining light is very low [8,9]. Such variance in the sensor responsivity with environmental temperature is radiometer component- and wavelength-dependent [29]. Indeed, we have observed that the sensor dark dependence on temperature is conditioned by the material used to build the sensor container, i.e., aluminum or polyether-ether-ketone (hereafter PEEK). Laboratory experiments have confirmed this temperature dependence for radiometers to be deployed in Arctic waters [30] and showed differences between those made in aluminum and PEEK across a wide range of ambient temperatures (see Supplementary Materials Section S1). Second, the sensors’ dark measurements may drift after several years of float operation. Radiometers mounted on Argo floats have not been equipped with mechanical shutters that acquire along cast dark measurements during daylight profiles, mainly due to relevant power consumption. We thus evolved the initially established sampling protocol towards the acquisition of reference night profiles and dark measurements at the 1000 dbar parking depth over the whole float lifetime in order to characterize, quality-control and solve these sources of variability in the sensor response.

As for all Argo physical and biogeochemical variables, radiometry quality-control (QC) must be provided in real-time (RT) and delayed-mode (DM). The RT-QC is mainly devoted to operational oceanography (e.g., assimilation in forecast models of ocean state) and consists in a number of automatic procedures that target the evaluation of a single profile at a time and QC data distribution within 12 h from sampling. The DM-QC aims to make data available within 12 months from the acquisition, after human control and exploiting all measured profiles together [31]. The resulting DM-QC dataset is expected to have the highest quality requested for scientific analysis and, ultimately, for climate studies. The RT-QC procedure for radiometry, accepted by the Argo Data Management Team, aims to check and flag measurements outside the range of expected values [32]. Alternatively, Organelli et al. [28] have proposed a near-real-time methodology detecting environmental signals in radiometric profiles due to clouds and wave focusing near surface, that is dedicated to bio-optical and remote-sensing applications (i.e., calm sea and uniform sky conditions during the measurement [33]). No DM-QC for radiometric data, as well as methods to characterize and solve sensor dark dependency on temperature and drift have been implemented yet. 

Here, we will exploit the global array of floats equipped with radiometers to develop and assess a DM-QC procedure that aims to correct the effect of changes in environmental temperature on BGC-Argo radiometric dark signals according to the material used to build the instrument, and account for sensor dark drift with time (hereinafter referred to as aging).

Following Equation (1) we convert digital counts (DC) to irradiance (units of W m^−2^ nm^−1^) and PAR (units of µmol photons m^−2^ s^−1^) values:

(1) $$E_{d}\left(\lambda \right)=Im\left(\lambda \right)*a_{1}\left(\lambda ,T_{s},t\right)*\left(DC\left(\lambda \right)-a_{0}\left(\lambda ,T_{s},t\right)\right)$$

this study will focus on the correction of the effects of the time, *t*, and of the sensor internal temperature, $T_{s}$, on the $a_{0}$ calibration coefficient for each band of each sensor (i.e., the dark signal), as the temperature dependency of the calibration coefficient $a_{1}$ has been found to be negligible [34]. *Im* is the immersion coefficient, fixed for each band. We will discuss procedure performance and show examples for a variety of trophic and illumination conditions encountered across the global ocean. Finally, we will present advantages, limitations and recommendations for the method. We anticipate the proposed methodology and the recommended sampling protocol will open the door to the operational distribution of the highest quality Argo radiometric profiles to the international oceanographic community. All symbols and abbreviations used here are listed in the nomenclature list given below.

## 2. Materials and Methods

### 2.1. The Biogeochemical-Argo Database

Biogeochemical-Argo data used to develop and assess the DM-QC procedure for radiometric profiles were acquired by 55 no longer profiling PROVOR-CTS4 floats, for a total of 12,867 measured radiometry profiles. This fleet has operated since 2012 across a variety of trophic environments and regional seas (Figure 1). All floats were configured and deployed according to standard procedures [35]. The data were downloaded from the Coriolis Global Data Assembly Center (GDAC) and stored in the Argo B and trajectory files (ftp://ftp.ifremer.fr/ifremer/argo (accessed on 1 November 2020)).

Floats were programmed to drift at a parking depth of 1000 dbar and acquire vertical profiles up to the sea surface every 1 to 10 days. Pressure and water temperature data were collected every 2 s by a SBE-41 CP conductivity-temperature-depth sensor (Sea-Bird Scientific, Bellevue, WA, USA), and quality-controlled according to standard, internationally-accepted protocols [36]. *E_d_* at three wavelengths (380, 412 and 490 nm) and PAR measurements were acquired by an OCR-504 radiometer (Sea-Bird Scientific), without an internal temperature probe and configured with a different sensor for each channel [37]. Though all the floats were equipped with the same radiometer model, the thermodynamic properties of five instruments made with aluminum (i.e., 693 profiles), deployed between 2014 and 2018, were different from those made with PEEK (see Supplementary Materials Section S1).

Radiometric profiles were acquired in the upper 250 dbar, around local noon to reduce the impact of low solar zenith angles [33]. To develop specific correction procedures for the dark correction, which is known to be temperature-dependent [38,39], night profiles (i.e., solar elevation < 5°) were acquired across a similar temperature range as day profiles since 2014, but neither systematically nor homogeneously among all floats. Moreover, radiometric measurements were also acquired daily during the float drift at the 1000 dbar parking depth to evaluate any change in the instrument’s response with time. This was implemented mid-2014 for all floats but those deployed in the Baffin Bay (Arctic Sea). Hereafter, we will refer to radiometric data used to develop and assess the DM-QC control such as: (i) day profiles (high light and high temperature variability); (ii) night profiles (no or very dim light with high temperature variability); and (iii) drift measurements (no light and low temperature variability).

In the following sections, we will show that both the acquisition of night profiles and daily radiometric measurement at 1000 dbar represent key ancillary measurements to correct the sensor’s dark signal and develop the most accurate DM-QC procedure. However, since in the Coriolis GDAC there are additional 11350 profiles acquired by 76 no longer profiling floats without sufficient ancillary night profiles or drift measurements acquired for longer than 80% of the float lifetime (Table 1), we have developed specific DM-QC procedures for those floats that are presented in the Supplementary Material Section S2. Hence, the following sections will only focus on the best possible DM-QC method that we recommend for future BGC-Argo radiometry deployments.

### 2.2. Reconstruction of the Sensor Internal Temperature

The thermodynamics response of the sensor is not instantaneous (see Supplementary Materials Section S1), thus the radiometer internal temperature must be reconstructed to develop the DM-QC procedure. Following laboratory experiments (see Supplementary Materials Section S1), the internal temperature $T_{s}$ at which the sensor operates was modeled using a delay first-order differential equation:

(2) $$\frac{1}{k}\frac{dT_{s}}{dt}\left(t\right)=T_{w}\left(t-\Delta t\right)-T_{s}\left(t\right)$$

where $T_{w}$ is the temperature of the surrounding water; *k* and Δ*t* are empirically estimated coefficients which represent the physical characteristics of the radiometer (Table 2).

To integrate Equation (2) along the entire profile, the following assumptions were made:

1) $T_{s}$ = $T_{w}$ at the bottom of the profile. All floats spend at least one day at 1000 dbar before profiling. Thus, when the float starts acquiring measurements, the sensor temperature is at the equilibrium with the environment (1⁄*k* + Δ*t* << 1 day);
2) The ascending speed of the float, *c*, is assumed to be constant, thus *c* = 0.1 dbar s^−1^. We analyzed 27,000 profiles from 165 PROVOR CTS-4 Argo floats, and found that 91% of the profiles showed an average ascending speed ranging between 0.08 dbar s^−1^ and 0.12 dbar s^−1^ (Figure 2). A sensitivity test on correction of E_d_(490) for the float WMO 6901654 revealed that, when using 0.08 and 0.12 dbar s^−1^ instead of 0.1 dbar s^−1^, the corrected E_d_(490) values change by at most 1.7 × 10^−5^ W m^−2^ nm^−1^, with 95% of the measurement points vary by less than 5.3 × 10^−6^ W m^−2^ nm^−1^. This observed variability is consistent with the manufacturer-established sensor noise of 2.5 × 10^−5^ W m^−2^ nm^−1^ [37].

We then introduce $T_{s}^{*}$ which is $T_{s}$ delayed by Δ*t*. This allows Equation (2) to be rewritten as an ordinary differential equation:

(3) $$\frac{1}{k}\frac{dT_{s}^{*}}{dt}=T_{w}-T_{s}^{*}$$

with:

(4) $$T_{s}^{*}=T_{s}\left(t+\Delta t\right)$$

Temperature is measured along a discrete axis of corresponding pressure measurements. We numerically integrate Equation (3) along this discrete axis with index 0 corresponding to the deepest (and first) measurement. We also introduce $t_{n}$, i.e., the time at which each measurement is taken, with $t_{0}$ = 0, and $P_{w_{n}}$ which is the pressure measurement associated to $T_{w_{n}}$.

From Assumption 1 described above:

(5) $$T_{s_{0}}^{*}=T_{s_{0}}=T_{w_{0}}$$

Equation (3) can be discretized as:

(6) $$T_{s_{n}}^{*}=T_{s_{n-1}}^{*}+k*\left(t_{n}-t_{n-1}\right)*\left(T_{w_{n-1}}-T_{s_{n-1}}^{*}\right)$$

Using Assumption 2, we can express:

(7) $$t_{n}=c^{-1}*\left(P_{w_{0}}-P_{w_{n}}\right)$$

so that Equation (6) becomes:

(8) $$T_{s_{n}}^{*}=T_{s_{n-1}}^{*}+\frac{k}{c}*\left(P_{w_{n-1}}-P_{w_{n}}\right)*\left(T_{w_{n-1}}-T_{s_{n-1}}^{*}\right)$$

Equation (8) can be computed to obtain $T_{s_{n}}^{*}$ for each $P_{w_{n}}$ value. The pressure axis $P_{s_{n}}$ is then defined as:

(9) $$P_{s_{n}}=P_{w_{n}}+c*\Delta t$$

so that for each *n*, $T_{s_{n}}$ is equal to $T_{s_{n}}^{*}$ when $T_{s_{n}}$ values are associated to the pressure axis $P_{s_{n}}$.

The final step is to interpolate $T_{s_{n}}$ to retrieve $T_{s}$ values that correspond to the pressure axis of radiometric measurements. 

To reconstruct the sensor internal temperature for radiometric measurements acquired during the float drift at the 1000 dbar parking depth, the model described by Equations (2)–(9) could not be applied because of the low frequency of drift measurements and the inapplicability of Assumption 2. In this case, because water temperature changes slowly during the drift of the float, and the float spends at least one day at those given depth and temperature, the closest (in time) water temperature measurement to the radiometry sampling was selected as the corresponding $T_{s}$.

## 3. Protocols for the Correction of Aging and Temperature Dependence of the Dark Signal

### 3.1. Theoretical Framework

The measured irradiance $E_{d_{meas}}$ is described as a function of the real irradiance $E_{d_{real}}$, the sensor internal temperature $T_{s}$, the time *t*, and the sensor random normal noise *ε*:

(10) $$E_{d_{meas}}=F\left(E_{d_{real}},T_{s},t\right)+\varepsilon$$

We assumed that:

(11) $$E_{d_{meas}}=h\left(T_{s},t\right)*E_{d_{real}}+f\left(T_{s}\right)+g\left(t\right)+\varepsilon$$

where *h* is the slope error introduced by the temperature effects and aging, *f*($T_{s}$) and *g*(*t*) are the dark errors introduced by the sensor temperature and aging respectively, which are assumed to be independent from one another. 

For night profiles and drift measurements, the float is in the dark so that $E_{d_{real}}$ is assumed equal to 0. Equation (11) is thus modified to:

(12) $$\mathrm{For}\;\mathrm{night}\;\mathrm{profiles},\;E_{d_{meas}}=0+f\left(T_{s}\right)+g\left(t\right)+\varepsilon$$

(13) $$\mathrm{For}\;\mathrm{drift}\;\mathrm{measurements},\;E_{d_{meas}}=0+f\left(T_{s}\sim constant\right)+g\left(t\right)+\varepsilon$$

In Equation (13), we indicate that the water temperature variations at the 1000 dbar parking depth are relatively small, which means $T_{s}$ can be considered as near constant. This also means that drift measurements at 1000 dbar parking depth can be used to estimate the sensor’s dark aging *g*(*t*) almost independently from changes in the environmental temperature. This estimated *g*(*t*) is then needed in Equation (12) to estimate the sensor’s dark temperature dependency *f*($T_{s}$) using night profiles, which are acquired over a larger range of temperatures than drift measurements. This is the rationale to estimate *g*(*t*) and perform the correction for sensor dark aging before the estimation of *f*($T_{s}$) and the correction of the sensor temperature-dependence.

### 3.2. Overview of the Procedure

The overall quality-control procedure includes five consecutive steps, which will be described in the following sections, and are the same both for *E_d_*(λ) and PAR: (i) Visual quality control; (ii) Correction of the sensor aging; (iii) Correction of the sensor temperature-dependence; (iv) Error estimation; and (v) Assignment of quality flags.

The overview of the whole procedure to correct for aging and then temperature-dependence of the dark sensor is shown in Figure 3. After the visual check, the workflow starts with the computation of a multiple linear or linear-quadratic regression that must be visually checked by the DM operator before applying the aging correction to all measured profiles of a given float. We remind that, for BGC-Argo DM-QC, the operator must use own scientific expertise and provide critical inputs to evaluate the correction results. If the correction for the aging does not yield satisfactory results, the DM operator may move to the following step. This is recommended for floats with short lifespan.

Corrected profiles are then adjusted for the temperature-dependence by computing linear regressions on night profiles. The linear regression must be visually checked by the DM operator before applying the correction to all measured profiles of a given float. The DM operator must thus evaluate that the temperature range covered by night profiles is representative of the temperature variability encountered by the float over the whole lifetime, as well as the regression fit to the data. If the method does not yield satisfactory results, the DM operator abandons the quality control of that float. An example of unsatisfactory linear regression is shown in Supplementary Material Section S3. If the correction is successful the error associated to each measurement is estimated and quality flags are assigned.

#### 3.2.1. Visual Quality Control

According to the standard Argo procedures [36], the DM-QC includes a preliminary visual check, profile by profile, made by the operator before the application of automatic correction routines. Thus, each data point within the profile is ultimately assigned one of the standard Argo QC flags: “1” for good data, “2” for probably good data; “3” for probably bad data; and “4” for bad data. Both flags 1 and 2 will be used to correct sensor’s dark aging and temperature dependence as described here below.

Practically, the visual check starts from the evaluation of RT-QC radiometry data [32]. The DM operator first evaluates if RT-QC Flag “3” measurements must be confirmed as bad or upgraded to “1” or “2”. Then, the operator visually detects any obvious outlier along the profile which is not related to environmental signals due to clouds and wave focusing/defocusing. The outliers are assigned to Flag “3” and “4” depending on the DM operator’s confidence. Radiometric measurements flagged as “3” and “4” are not further evaluated and are excluded from the following QC steps.

#### 3.2.2. Correction of the Sensor Dark’s Aging

In the following section, the protocol to correct the sensor dark’s aging which is based on the use of drift measurements is presented. Outliers are first removed from drift measurements and are defined as any value falling outside of the range between [1st_quartile–1.5*(3rd_quartile–1st_quartile)] and [3rd_quartile + 1.5*(3rd_quartile–1st_quartile)].

Following Equation (13), $E_{d_{meas}}$ is equal to 0 and $T_{s}$ at 1000 dbar shows relatively low variance. However, this small variance can still have a visible impact on the drift data (Figure 4). Apart from deviations due to temperature, the sensor aging most often appears as a linear function of time. Thus, *g*(*t*) is estimated by applying a multiple linear regression model of $E_{d_{meas}}$ as a function of *t* and $T_{s}$:

(14) $$E_{d_{meas}}^{*}=Ad+Bd*T_{s}+Cd*t$$

Subsequently, the DM operator must visually check the resulting fit from Equation (14) by estimating *E_d_* at a reference temperature which has been set to 5 °C (Figure 5):

(15) $$E_{d_{5c}}=E_{d_{meas}}-Bd*(T_{s}-5)$$

and:

(16) $$E_{d_{5C}}^{*}=Ad+5Bd+Cd*t$$

Because the aging may change sign and/or intensity over time (e.g., *E_d_*(412) in Figure 4), the DM-QC operator may not be satisfied with the results of the linear fit in Equation (14). In such a case, the operator may decide to fit $E_{d_{meas}}$ by a quadratic function versus *t* and linear versus $T_{s}$ (*E_d_*(412) in Figure 5):

(17) $$E_{d_{meas}}^{*}=Ad+Bd*T_{s}+Cd*t+Qd*t^{2}$$

so that:

(18) $$g\left(t\right)=Ad+Cd*t+Qd*t^{2}$$

where *Qd* is 0 when the linear regression in Equation (14) is applied. It should be noted that Equation (18) includes the constant offset *Ad* from the bilinear regression. *Ad* is not mathematically required to compute *g*(*t*) because another coefficient will be computed when temperature correction is performed (see following sections). However, it is here included in order to allow the DM operator to run the procedure using realistic radiometric values.

The multiple linear model described by Equation (14) is able to correct for the small temperature variations found at the 1000 dbar parking depth. However, this temperature correction cannot be applied to the whole profiles because they span a large range of variability in temperature so that estimated coefficients from Equation (14) are not suitable. In addition, the estimation at a reference temperature of 5 °C allows the DM operator to visualize and evaluate, float by float, the goodness of the aging’s correction procedure. However, if the operator is still not satisfied with the proposed correction after visual check, we suggest to proceed with the temperature-dependence correction anyway and test the results. This is especially recommended for floats with a short lifespan.

#### 3.2.3. Correction of the Sensor Dark’s Temperature Dependence

In this section, the protocol to correct the sensor dark’s dependence on temperature which is based on the use of night profiles is presented. We recall that $E_{d_{real}}\;$ is assumed equal to 0 along the whole night profile, which covers a large variability in water temperature. As a first step, all night profiles collected by a single float are corrected for the sensor aging as described above. $E_{d_{night}}$ is then defined as:

(19) $$E_{d_{night}}=E_{d_{meas}}-g\left(t\right)=E_{d_{meas}}-Ad-Cd*t-Qd*t^{2}=f\left(T_{s}\right)+\varepsilon$$

Then, $E_{d_{night}}\;$ is linearly fitted as a function of the reconstructed sensor internal temperature $T_{s}$:

(20) $$E_{d}^{*}{}_{night}=At+Bt*T_{s}\;$$

Figure 6 shows an example of aging-corrected night profiles and regression analysis. It is important to note that some night profiles might be influenced by the moon and star light or acquired close to dawn and dusk. To remove such polluted data, the DM operator may select a pressure threshold.

Subsequently, the offset to correct for sensor darks’ dependence on temperature is expressed as:

(21) $$f\left(T_{s}\right)=At+Bt*T_{s}$$

The final correction to be applied to all 0–250 dbar profiles is finally expressed as:

(22) $$E_{d_{corr}}=E_{d_{meas}}-f\left(T_{s}\right)-g\left(t\right)$$

(23) $$E_{d_{corr}}=E_{d_{meas}}-At-Bt*T_{s}-Ad-Cd*t-Qd*t^{2}$$

(24) $$E_{d_{corr}}=E_{d_{meas}}-A-B*T_{s}-C*t-Q{*t}^{2}\;\left(24\right)$$

where *A = At + Ad*, *B = Bt*, *C = Cd*, and *Q = Qd*. It must be noted that the corrected irradiance $E_{d_{corr}}$ is not equal to $E_{d_{real}}$ (Equation (11)) as only the temperature and aging effects on the dark signal have been corrected. To equate $E_{d_{corr}}$ and $E_{d_{real}}$, *h*($T_{s}$,*t*) in Equation (11) must be assumed equal to 1.

#### 3.2.4. Error Estimation

Upon implementation of corrections presented above, the error associated with each measured value ($\sigma_{Ed}$) is estimated as the maximum value between the Noise Equivalent Irradiance (NEI) (as provided by the manufacturer), and the relative error (ER) multiplied by the corrected radiometry value $E_{d_{corr}}$:

(25) $$\sigma_{E_{d}}=\max\left(NEI_{E_{d}};ER_{E_{d}}*E_{d_{corr}}\right)$$

$NEI_{E_{d}}$ is the manufacturer’s NEI value of OCR-504 radiometers equal to 2.5 × 10^−5^ W m^−^^2^ nm^−1^ for all *E_d_*(λ) [37]. For PAR, $NEI_{E_{d}}$ was estimated by computing the maximum standard deviation observed for the dark values at the 1000 dbar parking depth corrected for any aging among a total of 34 selected floats. The resulting $NEI_{E_{d}}$ for PAR is equal to 0.03 µmol photons m^−2^ s^−1^. ER is 5% for PAR [40] and 2% for *E_d_*(λ) following previous calibration error estimations [41,42].

#### 3.2.5. Assignment of Quality Flags on Temperature Corrected Profiles

The DM-QC flags on sensor aging and temperature corrected profiles are assigned according to the following procedure:

- Recover the QC flags assigned with the visual QC. These profiles contain Flags “1”, “2”, “3” and “4”;
- Detect the dark values within corrected profiles applying successive Lilliefors tests (α = 0.01; ref. [28]), and assign Flag “2”;
- Change radiometry flags “3” or “4” due to visual QC to “4”;
- If pressure QC flag is “3” or “4”, radiometry flag is assigned as “4”;
- If $T_{s}$ cannot be reconstructed, the radiometry flag is assigned as “4”.

## 4. Performance of the DM-QC Procedure

The DM-QC procedure described above to correct for sensor dark changes with time and varying environmental temperature was tested over a total of 55 BGC-Argo profiling floats with ancillary night profiles and drift measurements acquired over more than 80% of the float lifetime. All these floats, operating across the globe, were equipped with OCR-504 radiometers and acquired 0–250 dbar E_d_ profiles at 380, 412 and 490 nm in addition to PAR. 

In Figure 7, we show examples of vertical profiles before and after correction for sensor’s dark aging and temperature dependence. The magnitude of the correction applied as represented by the A, B, and C parameters obtained through Equation (24), and its variability over the ensemble of floats whose sensor aging was corrected linearly are shown for each band in Figure S9 (Supplementary Materials Section S4). The distributions of the A, B, and C parameters were generally normal and, the impact of temperature on the sensor’s dark signal showed to be larger than the one due to the sensor’s aging. 

Examples of corrected profiles (Figure 7) encompass a variety of oceanic environments with diverse optical, trophic and biogeochemical conditions [4,20,43,44], thus showing applicability of the procedure at the global scale. In particular, the steps we set up for the DM-QC BGC-Argo radiometry (Figure 3) provide adjustments of specific features that characterize the profiles (Figure 7). First of all, all non-zero dark measurements at depth are shifted to zero or re-qualified as very low irradiance measurements that, otherwise, would have been disregarded. Indeed, the DM-QC procedure makes vertical profiles usable at greater depths so that biogeochemical, modelling, and optical applications can be enhanced. This is particularly relevant for permanently oligotrophic clear waters (e.g., mid-ocean gyres; Figure 7d) where sunlight around local noon can penetrate deeper than 250 dbar [8], or in productive high-latitude seas during wintertime where the underwater light field can expand down to 150 dbar (Figure 7j) and contribute to phytoplankton blooms [18].

Contrarily, in the upper part of the profile where irradiance values are the highest and aging and temperature issues are expected to have a negligible impact [8,28], the developed correction protocols do not determine significant changes in the measured values (Figure 7). In addition, the developed QC procedure does not affect the signature of the environmental signals such as those due to clouds and wave focusing/defocusing (Figure 7a). Such characteristics reinforce previously published scientific studies restricted to the first optical depth or the mixed layer [4,6,20], and joint applications with remote sensing observations [8,11]. Yet a newly generated radiometric database enhanced with sensor dark’s aging and temperature-dependence corrections will surely open to the possibility of re-analysis studies.

However, the applied DM procedure correctly resolves artificial features such as steps in the profiles due to a significant increase of the dark counts which respond to the sudden changes in water temperature (Figure 7g–i). The developed protocols remove these features and shift to zero dark values at depth, so that the resulting radiometric profiles show the monotonic decrease with depth as expected.

The DM-QC procedure we developed has been implemented over a total of 12,867 measured profiles each band. The procedure returned profiles that monotonically decreased as expected from theory and reached greater depths (Figure 7). A total of 11,824 profiles (from 47 floats), i.e., about 92% of the tested database for bands at 412 and 490 nm, and PAR was corrected (Figure 8). In the case of E_d_(380), correction was successful for 11,597 profiles (from 46 floats), i.e., 90% of the tested database. In particular, the DM successfully corrected profiles derived from 45 floats made with PEEK components (44 floats for E_d_(380)), and two floats with aluminum components. The uncorrected 227 E_d_(380) profiles (all from one float) were corrected with alternative procedures (see Supplementary Materials Section S2). The developed QC procedure demonstrated high and similar performances for all radiometric channels. This suggests strong potential to implement these DM-QC protocols to other wavelengths and, ultimately to hyperspectral radiometers. 

Regarding the remaining uncorrected 8 floats and 1043 radiometric profiles: 582 profiles from three floats (i.e., about 5% of the tested database) were corrected with alternative procedures specifically developed for the array of 76 floats with an insufficient number of night profiles or drift measurements (Supplementary Materials Section S2), while 461 profiles from five floats (i.e., about 4% of the tested database) were not corrected. Correction was made with alternative procedures when the ancillary data (most often night profiles) were not good enough to confidently apply the procedure described here, correction was abandoned when the alternative methods also failed.

Overall, the DM-QC procedure to correct the sensor dark signal systematically succeeded for all tested floats with at least four night profiles collected over the float lifetime (Figure 9). 

Nevertheless, the majority of floats had three or fewer associated night profiles over their lifetime, and the correction we implemented was still successful in most of those cases. As the average lifespan of a float is expected to be four years [3], our results thus implies that each float equipped with radiometers must acquire one night profile per year, preferably during moonless nights and when the temperature range between the surface and 1000 dbar parking depth is the largest.

## 5. Discussion and Conclusions

To quality-control the large amount of radiometric profiles acquired by BGC-Argo floats, real-time [32] and near real-time quality-control procedures [28] have been proposed. While the method proposed by Poteau et al. [32] was mainly verifying the range of measured values, Organelli et al. [28] proposed protocols for the qualification of radiometric profiles to specifically use in ocean optics science and remote sensing applications e.g., for the derivation of the diffuse attenuation coefficient K_d_ which is a key quantity for bio-optical and biogeochemical studies [4]. With this aim, their method was not focusing on the issues addressed here (i.e., sensor’s dark dependence on temperature and aging) but rather on how the environment (presence of clouds, wave focusing at the surface) drives departures of the profile with respect to an expected monotonic decrease of irradiance with depth. Moreover, the scientific exploitation of the quality controlled radiometric profiles according to Organelli et al. [28] was restricted to the upper layer (i.e., first penetration depth [45]), mainly because some inconsistencies likely due to sensor dark’s temperature-dependence issues were noticed in the deepest part.

The method proposed here offers a pragmatic way to identify and correct BGC-Argo radiometric profiles for sensor dark’s aging and temperature-dependence issues, by acquiring one night profile per year and daily dark measurements at the 1000 dbar parking depth. These new protocols will allow to extend the range of exploitable measurements and, ultimately, enhance their use among the international biogeochemical community. Yet, we also recommend a technological upgrade of radiometers installed on floats with a probe to directly monitoring the internal temperature at which the sensor operates, which has only been modelled so far.

We must notice that the joint use of the DM-QC method here proposed with the one presented by Organelli et al. [28] represents an opportunity to generate a unique high-quality and interoperable radiometric dataset free of clouds and wave focusing/defocusing. Given the potential for the BGC-Argo network to expand [2,46], it can be expected that the resulting dataset, potentially increasing in near real-time, would allow addressing or readdressing key topics of applications in ocean optics the investigation of which was up to now suffering from limited data availability. The quality of the data could be further enhanced when also the impact of instrument tilt on measured values as well as the effect of bio-fouling that can occur [8] will be taken into account.

Among these ocean optics science topics, the understanding of regional and seasonal variability of K_d_ with a higher degree of confidence must be refined along the water column [4]. Additionally, comparing such in-situ BGC-Argo float products with their satellite counterparts would allow the identification of the locations where bio-optical anomalies or nuances exist. This would represent a preliminary step to understand the causes of discrepancies and, as a consequence, possibly refine the retrieval algorithms for satellite products in some areas.

The possible derivation of radiometry with depth over the whole vertical dimension is expected to provide high resolution K_d_ profiles that will be useful to address the link between surface remotely-sensed properties and their vertical variability according to region and season. Such data could in turn allow to re-evaluate and possibly improve methods developed to retrieve the vertical profile of chlorophyll-a from simultaneous measurement of chlorophyll fluorescence and radiometry from floats, methods that were initially developed on a very small float dataset [47].

The improved accuracy of radiometric measurements with depth will also enhance their use across the biogeochemical and ecosystem model community. An improved accuracy is expected to support studies that assimilate irradiance data to model phytoplankton photosynthesis [26], especially at the most elevated depths where the deep chlorophyll maxima are observed and supported by small quantities of light.

When considering the DM corrected profiles over the whole tested database, the method we presented showed high and similar applicability for the three channels of downwelling irradiance as well as for PAR, thus suggesting potential applicability to hyperspectral radiometers. With the advent of future hyperspectral satellite missions [48], there is an increasing interest in in-situ hyperspectral optics. Profiling floats equipped with hyperspectral radiometers represent an especially cost-effective approach to evaluate satellite performances during the post-launching so-called commissioning phases (few months). Such technology would indeed allow the acquisition of numerous calibration/validation high-quality matchups in a limited period of time, provided that a significant fleet of dedicated floats [49] would be deployed in diverse environments with specific bio-optical status and atmospheric specificities. Additionally, hyperspectral measurements could possibly become a component of the standard BGC-Argo fleet offering the possibility to refine the detection and quantification of optically significant substances (phytoplankton communities, detritus, mineral substance, colored dissolved organic matter).

Finally, it should be noticed that with the increasing development of robotic observation systems, a fleet of sensors can now be deployed and operated globally which definitely will change our way to look at data and qualify them. Working with a dense dataset acquired from multiple-a priori identical and interoperable-instruments will indeed allow us to identify sensor issues that would be difficult to discover on a case-by-case analysis [43]. In this respect the BGC-Argo network represents a unique platform to help in improving sensor performances for the benefit of other observation systems.

## Figures and Tables

**Figure 1:**
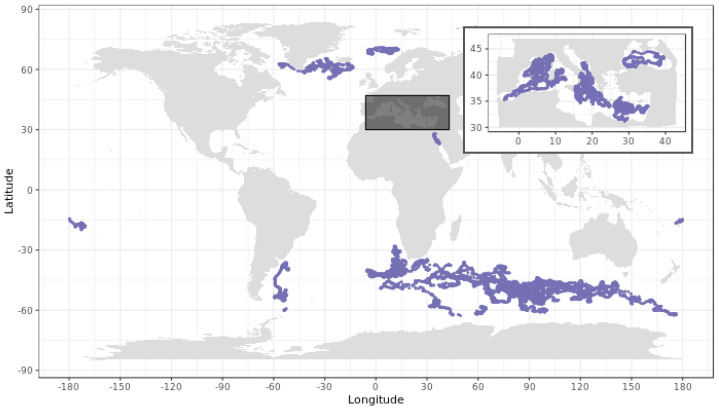
Sampled stations by the 55 profiling BGC-Argo floats considered in this study.

**Figure 2:**
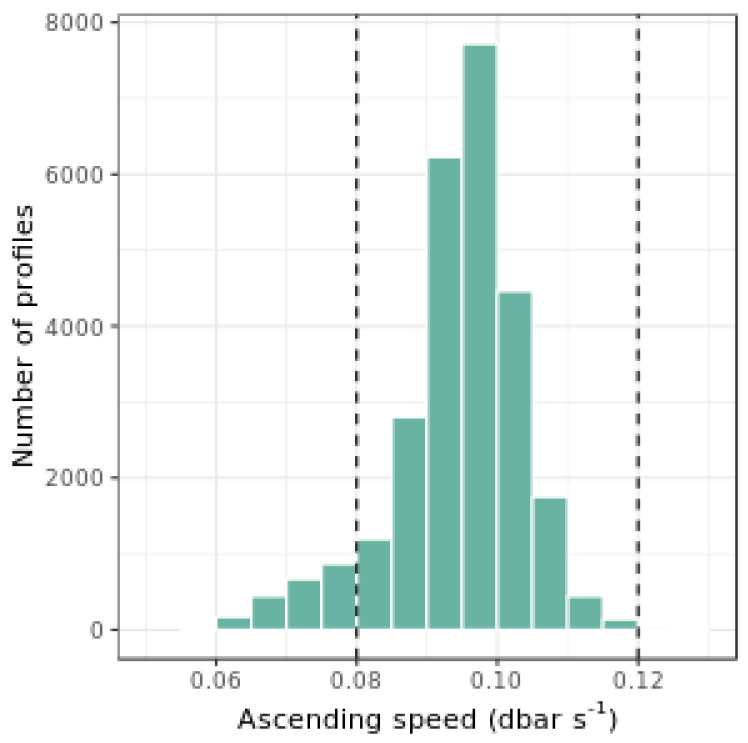
Histogram of the average float ascent speed for 27000 BGC-Argo radiometry profiles, reconstructed from the available time stamps in the trajectory profile. Vertical dashed lines indicate the two values used for the sensitivity test which interval includes 91% of tested profiles.

**Figure 3:**
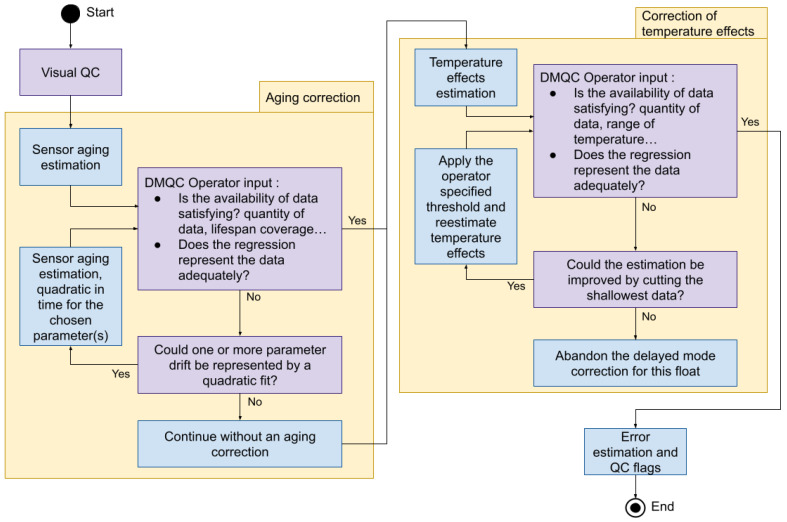
Flowchart of the QC procedure to correct radiometry for aging and temperature dependency.

**Figure 4:**
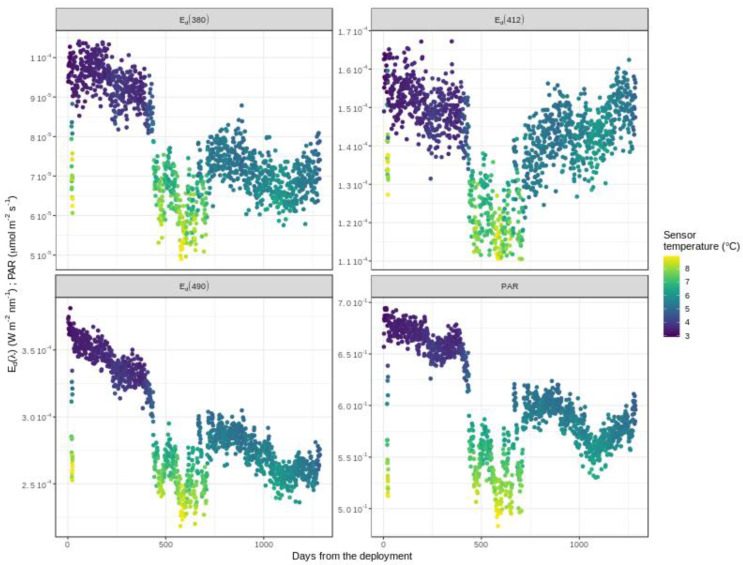
Radiometry drift measurements for E_d_(λ) and PAR as a function of time and temperature. Example is shown for the float WMO6901584.

**Figure 5:**
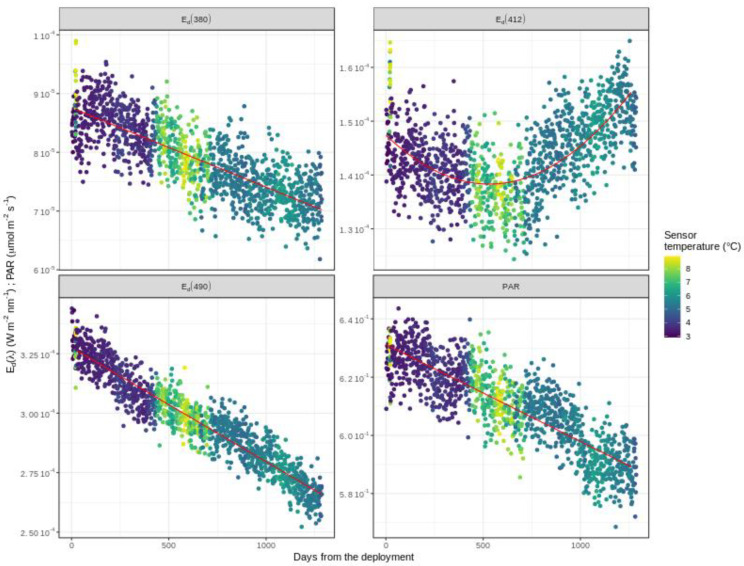
Radiometry drift measurements for E_d_(λ) and PAR as a function of time after estimation at a reference temperature of 5 °C. Solid line is the fit to all points. For this float, the fit is linear for all channels but E_d_(412). Example is shown for the float WMO6901584.

**Figure 6:**
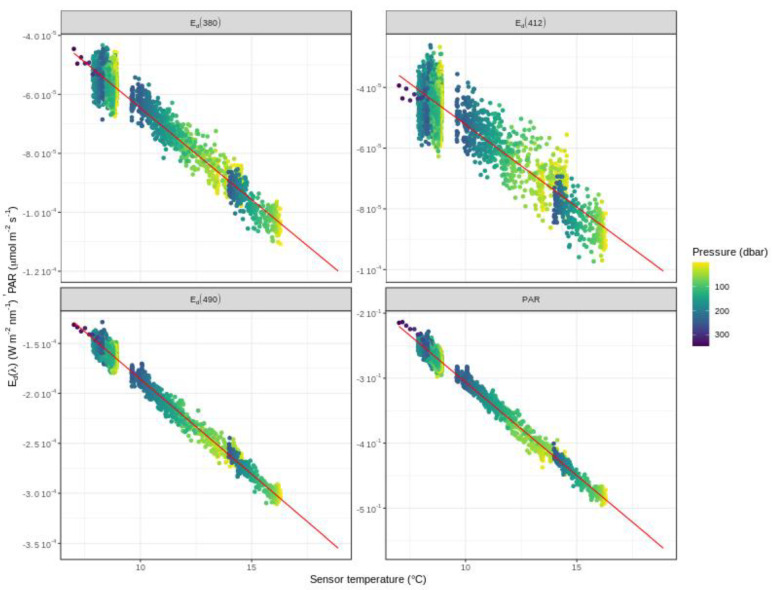
Radiometry night profiles of E_d_(λ) and PAR as a function of sensor internal temperature $T_{s}$. Dots are colored according to pressure. Solid red line is the fit to all points, and is extrapolated to cover the entire range of temperature encountered by the float during the whole lifetime. Prior to computing the linear regression, night profiles have been corrected for any sensor aging. Example is shown for the float WMO6901584.

**Figure 7:**
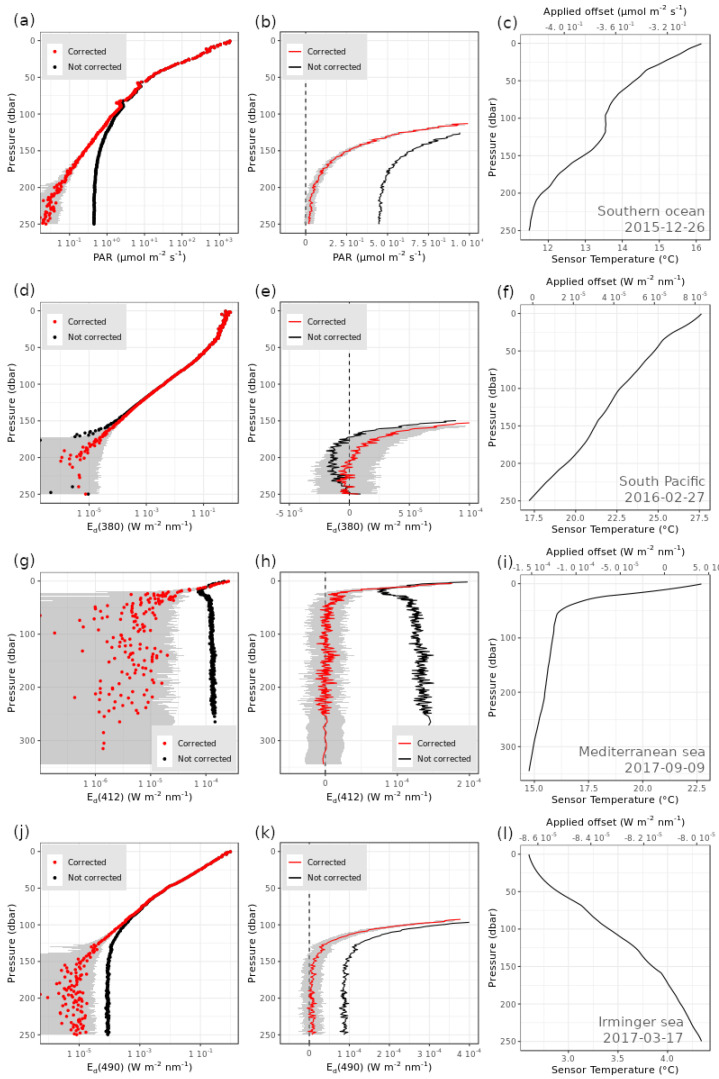
Examples of radiometry profiles before and after DM-QC: Left) profiles are shown in a semi-log scale; Centre) profiles are shown in a linear scale; Right) the reconstructed sensor internal temperature $T_{s}$ is shown (Equations (2)–(9)). Examples derive from four BGC-Argo floats deployed in oceanic regions characterized by diverse trophic and optical regimes: (**a**–**c**) Southern Ocean; (**d**–**f**) South Pacific subtropical gyre; (**g**–**i**) Mediterranean Sea; (**j**–**l**) North Atlantic subpolar gyre—Irminger Sea.

**Figure 8:**
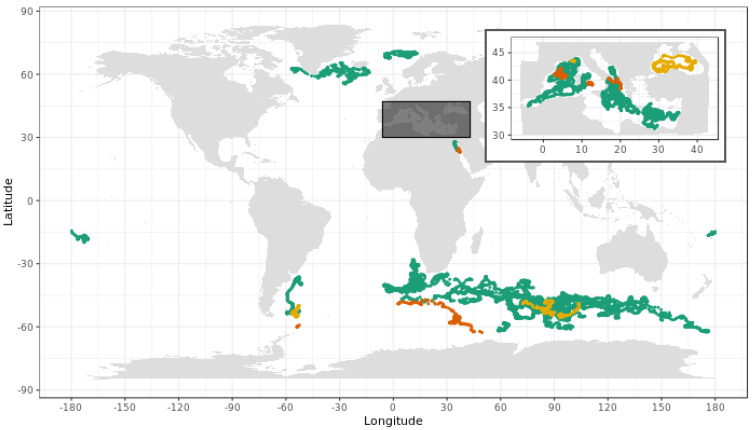
Radiometry profiles acquired by the 55 BGC-Argo floats with ancillary night profiles and drift measurements. Green dots: successfully corrected profiles with the DM-QC procedure; Orange dots: uncorrected profiles; Yellow dots: profiles corrected with alternative methods (see Supplementary Materials Section S2).

**Figure 9:**
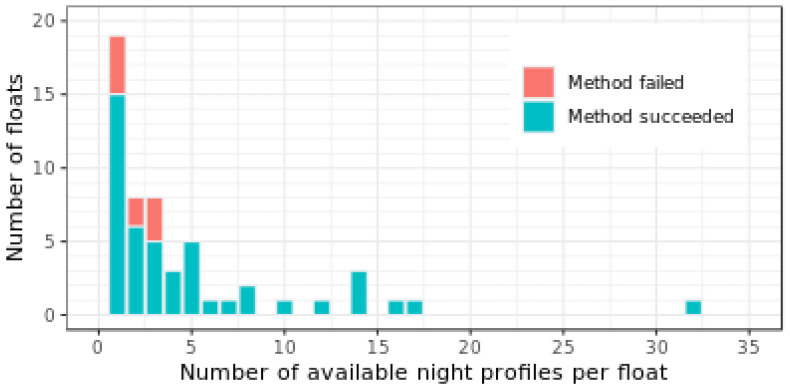
Number of floats with dark measurements successfully corrected for the four radiometric channels as a function of available night profiles.

**Table 1:** Availability of night profiles and daily drift measurements for the 55 and 76 BGC-Argo floats.

OCR 504 Model	Drift Acquired for > 80% of the Float Lifetime		Drift Acquired for ≤ 80% of the Float Lifetime		Total
	Night Profiles	No Night	Night Profiles	No Night	
PEEK	50	10	32	17	109
Aluminum	5	1	9	7	22
All	55	11	41	24	131

**Table 2:** Parameters used to reconstruct the sensor internal temperature *T_s_* according to the material of the radiometer components.

OCR 504 Model	k	Δ*t*
PEEK	0.2 min^−1^	1 min
Aluminum	0.44 min^−1^	0.25 min

## Data Availability

The data used in this study are openly available at https://doi.org/10.17882/42182 (accessed on 13 September 2021).

## Supplementary Materials

The following are available online at https://www.mdpi.com/article/10.3390/s21186217/s1.

## Computer Code and Software

The computed code to implement the protocols presented in this study is openly available at https://github.com/qjutard/radiometry_QC (version 1.03, accessed on 13 September 2021).

## Nomenclature

Symbol	Definition
$E_{d}$	Downwelling irradiance
PAR	Photosynthetically Available Radiation
Im	Immersion coefficient
$a_{0};\;a_{1}$	Calibration coefficients
DC	Dark counts
t	Time
$T_{s}$	Sensor internal temperature
k	Rate of change of the sensor temperature
$T_{w}$	Water temperature
Δt	Response delay of the sensor temperature to the water temperature
c	Ascending speed of floats (assumed constant)
$T_{s}^{*}$	Sensor temperature delayed by Δt
$T_{s_{n}}$	Discretized sensor temperature
$T_{w_{n}}$	Water temperature measurements, sorted from the deepest to the shallowest
$T_{s_{n}}^{*}$	Discretized delayed sensor temperature, follows the water temperature measurements axis
$t_{n}$	Discretized time corresponding to water temperature measurements
$Pw_{n}$	Pressure measurements associated to water temperature measurements
$Ps_{n}$	Pressure axis associated to $T_{s_{n}}$
$E_{d_{meas}}$	Measured irradiance
$E_{d_{real}}$	Real irradiance that would be obtained with a perfect sensor
*h*	Slope error introduced by the temperature and aging effects
ε	Sensor noise
$f\left(T_{s}\right)$	Error offset caused by the sensor temperature being different from calibration
g(t)	Error offset caused by sensor aging over time
$E_{d_{meas}}^{*}{}_{}$	Measured irradiance, fitted to $T_{s}$ and t
Ad, Bd, Cd, Qd	Coefficients in the fit of drift measurements to $T_{s}$ and t
$E_{d_{5C}}$	Measured irradiance in drift, projected on the $T_{s}=5$ °C plane along the $E_{d_{meas}}^{*}{}_{}$ fit
$E_{d_{5C}}^{*}$	$E_{d_{meas}}^{*}{}_{}$ projected on the $T_{s}=5$ °C plane along the $E_{d_{meas}}^{*}{}_{}$ fit
$E_{d_{night}}$	Irradiance measurements in night profiles, corrected for sensor aging
$E_{d_{night}}^{*}$	$E_{d_{night}}$, fitted to $T_{s}$
At	Coefficients in the fit of night measurements to $T_{s}$
Bt	
$E_{d_{corr}}$	Irradiance corrected for the effects of temperature and aging on the dark signal
A, B, C, Q	Coefficients in the full expression of the irradiance correction
$\sigma_{E_{d}}$	Error associated to $E_{d}$
$NEI_{E_{d}}$	Noise Equivalent Irradiance
$ER_{E_{d}}$	Relative Error
