# Supplementary material for: Correction of Biogeochemical-Argo Radiometry for Sensor Temperature-Dependence and Drift: Protocols for a Delayed-Mode Quality Control
Source: Sensors (Basel). 2021 Sep 16;21(18):6217. doi: 10.3390/s21186217 (PMC8473398; doi:10.3390/s21186217)
Supplement: Supplementary file 1 [file sensors-21-06217-s001.zip › sensors-1301062-supplementary.pdf]

Supplementary Material

Correction of Biogeochemical-Argo radiometry for sensor temperature-dependence and drift: protocols for a delayed-mode quality control

Jutard Q. et al.

Table of Contents

Section 1. Laboratory experiments to characterize the temperature-dependence of the OCR504 dark signal (pages 2–7)
Section 2. Alternative procedures to correct dark aging and temperature dependence (pages 8–10)
Section 3. Example of failure for correction protocols using drift and night measurements (page 11)
Section 4. Magnitude and variability of the dark correction (page 12)
Section 5. References (page 13)

Section 1. Laboratory Experiments to Characterize the Temperature-Dependence of the OCR504 Dark Signal

1.1 Sensors

Several sensors are experimented in this study, one is an OCR504 (called as OCR504-Solo, S/N: 507) with an internal temperature probe (resolution of 0.5°C, it is calibrated with an SBE39 in our lab), and others are RemA (listed in Table S1), a sensor package which integrates an OCR504 (without temperature probe) and an ECOPUCK sensor (a chlorophyll-fluorometer, a CDOM fluorometer and a backscattering sensor), most commonly used on the BioGeoChemical-Argo floats.

Table S1. Serial numbers of tested OCR504s, corresponding RemA serial numbers (S/N) and float names.

OCR504 S/N	RemA S/N	Float WMO	Temperature Range	Pressure housing material	Replicates
	014		4°C to 20°C	Aluminum	3
	161		4°C to 20°C	Plastic	2
	162		4°C to 20°C	Plastic	2
	163		4°C to 20°C	Plastic	2
345	140	6902666	4°C to 30°C	Plastic	1
346	141	6902669	4°C to 30°C	Plastic	1
353	139	4901804	4°C to 30°C	Plastic	1
375	157	4901805	4°C to 30°C	Plastic	1
388	164	6902670	4°C to 30°C	Plastic	1
389	165	6902671	4°C to 30°C	Plastic	1
390	166	6902829	4°C to 30°C	Plastic	1
507	/	/	5°C to 30°C	Plastic	1

At each band, the OCR504 records a digital count (DC) value with the sampling rate of 1.1 Hz (0.91 sec). The DC value could be converted to the irradiance value (units of μW cm^{-2} nm^{-1} for 380 nm, 412 nm and 490 nm) and PAR value (units of μmol photons m^{-2} s^{-1}), through a linear Equation:

$$E_d(\lambda) = Im(\lambda) * a_1(\lambda) * (DC(\lambda) - a_0(\lambda)) \quad (1)$$

Here, *Im* is the immersion coefficient, fixed for each band and independent of instrument (1.161, 1.368, 1.365 and 1.359 for four bands, respectively). It is only used when the sensor is immersed in water and becomes 1 when the sensor is in air. a_1 and a_0 represent the calibration coefficients for each band of each sensor. The sensitivity provided by the producer is 0.0025 μW cm^{-2} nm^{-1} for E_d(380 nm, 412 nm, 490 nm), and 0.01 μmol photons m^{-2} s^{-1} for PAR.

1.2 Laboratory Experiments

The experiment procedures follow:

1) Prepare two thermostats, one with water at 5°C and the other at 30°C (RemA were experimented from 4°C to 20°C/30°C, listed in Table S1);
2) Cover the all four receivers of experimented OCR504 (Solo or on RemA) with black tape;
3) Immerse it into the water of the thermostat with 5°C;
4) Wait for more than 50 mins after the water temperature reached 5°C, to make sure that the sensor internal temperature reaches the same temperature as the water (i.e., 5°C);
5) Start to record the dark currents of OCR504;
6) Move it into the thermostat with 30°C, and note the moving time;
7) Keep recording OCR504 more than 50 mins, to make sure that the sensor internal temperature reaches the same temperature as the water (i.e., 30°C).

1.3 Results of Laboratory Experiment on the Temperature Effect

Based on the laboratory experiment on OCR504 Solo, it is confirmed that, the dark current temperature effect of OCR504 radiometer includes three processes: a dark current response to sensor temperature, the heat conduction between external (water) temperature and internal (sensor) temperature, and a delay time of heat conduction.

1.3.1 Dark Current Response to Sensor Temperature

The dark current of the OCR504 radiometer responds to the sensor internal temperature, rather than the water temperature. Figure S1 shows the relationships between sensor temperature (T_s) and the dark currents (*Dark*) of four channels, which can be described with a linear relationship, as follows:

$$Dark(\lambda) = A(\lambda) + \mathrm{B}(\lambda) * T_s \quad (2)$$

Here, λ means different channels (wavelengths); A and B are constant coefficients for each channel: A is the Dark value at 0°C; and B is the change of Dark value per degree. For the radiometer presented in Figure S1, the coefficient B is positive for 380 nm and the broadband PAR channel, but negative for 412 nm and 490 nm, suggesting that the dark current may increase or decrease with temperature increasing, and its absolute value represents the sensitivity to temperature. For OCR504 Solo, B is largest at 380 nm, reaching 0.00581 μW cm^{-2} nm^{-1} $°C^{-1}$, which means that even if the sensor temperature varies by 1°C, the response of Dark currents at this channel will be more than twice the stated sensor sensitivity.

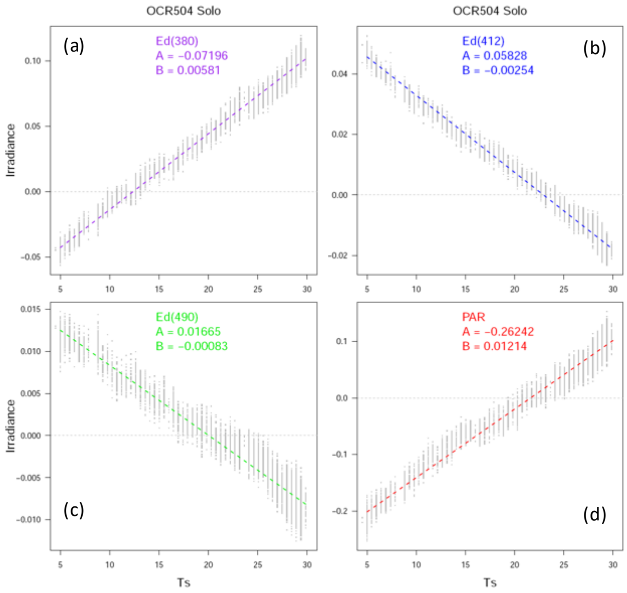

Figure S1. Example of OCR504 dark irradiance readings as a function of internal temperature (T_s), fromOCR504 s/n 00021. Colored dashed lines show linear regressions and colored text show regression coefficients corresponding to Equation (2). Units of μW cm^{-2} nm^{-1} for $E_d(\lambda)$ and μmol photons m^{-2} s^{-1} for PAR.

1.3.2 Delay Effect

Owing to lack of sensor internal temperature measurement for OCR504 radiometers equipped on BGC-Argo floats, it is important to understand the relationship between sensor temperature (T_s) and water temperature (T_w) in order to correct the sensor Dark values. Figure S2a displays a time series of T_s after immersing the sensor into a thermostat set at 30°C. Note that, T_w is maintained at 30°C during this period. The heat conduction process is very clear, T_s increases gradually from 5°C to 30°C in about 25 mins, however, it is interesting that the sensor internal temperature does immediately not follow a simple heat conduction function (See Section 1.3.3). Rather, there is a delay of ~ 60 secs before a response is observed. This delay effect is probably due to the capacitance loading, self-heating, interactions of multiple parts, or physical changes especially in connectors or solder whiskers. The delay effect can be expressed as

$$T_w^*(t) = T_w(t + \Delta t) \quad (3)$$

Here T_w^* is the delay-effected water temperature which affects the sensor temperature immediately, Δt represents the delay time (~ 60 secs).

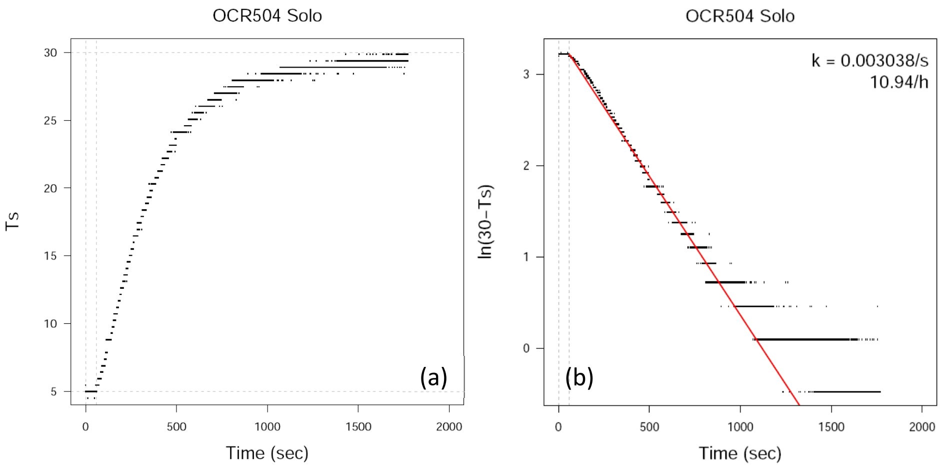

Figure S2. Example internal temperature (T_s) response to an abrupt change in external temperature. Data are from OCR504 s/n 00021, equilibrated at 5°C and immersed in 30°C water: (**a**) X-axes show time since immersion and y-axes show T_s; and (**b**) the natural logarithm of the difference between ambient and internal temperature. Red line in panel (**b**) shows regression used to estimate heat transfer coefficient k, shown in text within the figure.

1.3.3 Heat Conduction

After removing the delay period, the evolution of T_s then can be described with the classic heat conduction equation (Equation 4):

$$dT_s/dt = k * (T_w^* - T_s) \qquad (4)$$

Two temperatures are related by a response coefficient (k), proportional to the thermal conductivity of the materials used to build the sensor's container. Due to the constant T_w during this experiment, T_w^*=T_w =30°C, it can be expressed as:

$$dT_s/dt = k * (30 - T_s) \qquad (5.1)$$

Equation (5.1) can be re-written as:

$$d(30 - T_s)/dt = -k * (30 - T_s) \qquad (5.2)$$

then we obtain:

$$k = -d(\ln(30 - T_s))/dt \qquad (5.3)$$

Equation (5.3) suggests that the value $\ln(30 - T_s)$ will linearly vary with time (t), and the slope of linear relationship is k, as shown in Figure S2b. Applying a linear regression analysis, k is retrieved as 0.003038/s for this sensor, or 0.1823/min, which means that if the difference between T_s and T_w^* is 30°C, the change rate of T_s is 0.09°C/s.

Combining all three processes, the temperature effect on the dark current can be modeled as shown in Figure S3, and thus corrected, based on two variables, water temperature ($T_w(t)$) and sensor temperature at the initial time ($T_s(t_0)$, which can be assumed to be $T_w(t_0)$ if T_w is approximately stable before the initial time for a long time, e.g., at least 30 mins), and four parameters, i.e., A(λ), B(λ), k and Δt. Among them, A(λ) and B(λ) are sensor-dependent parameters, thus have to be corrected channel by channel and sensor by sensor; while k and Δt are material-dependent parameters, thus assumed to be constant

for all radiometers of same type (same material and same circuit design).

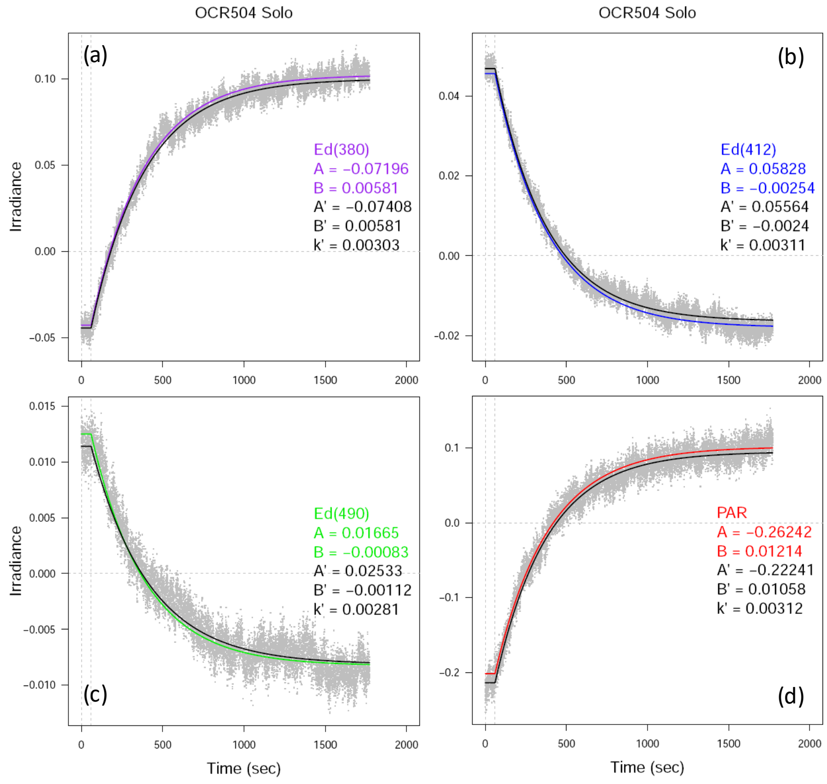

Figure S3. Example of OCR504 dark irradiance readings as a function of time following the immersion experiment shown in Fig. S2, from OCR504 s/n 00021. Colored curves show fits of Equation (6) with k fixed at the estimate determined in Fig. S2. Black curves show fits of Equation (6) allowing k to vary. Units of μW cm^{-2} nm^{-1} for $E_d(\lambda)$ and μmol photons m^{-2} s^{-1} for PAR.

1.3.4 Determination of k and Delay Time of RemA

So far, most radiometers on BGC-Argo floats are integrated with ECO sensors (i.e., RemA Type); few are OCR504-Solo. The extra thermal mass of the RemA unit could potentially affect the thermal kinetics of the integrated OCR504, so the laboratory tests described in section 1.2 were repeated for eleven RemA. Because T_s was not measured, k and Δt were estimated (at each channel for each sensor), by fitting an exponential (Equation (6)) directly to each sensor dark timeseries:

$$D(t) = \begin{cases} D_0, & \text{if } t \leq \Delta t \\ D_{eq} + (D_0 - D_{eq})e^{-k(t-\Delta t)}, & \text{if } t \geq \Delta t \end{cases} \quad (6)$$

where t is the time since immersion in the second thermostat, D_0 is the dark value at the temperature of the initial thermostat, and D_{eq} is the equilibrium dark value at the temperature of the second thermostat. Before fitting Equation (6), D_0 (Figure S4, horizontal dashed lines) was estimated as the mean value of D for the 30 s prior to immersion in the second thermostat (Figure S4, magenta lines), and D_{eq} (Figure S4, solid horizontal line) was estimated as the mean value of D the ten minutes starting 25 min after immersion (Figure

S4, green lines). Equation (6) (Figure S4, red lines) was then fit to *D(t)* (Figure S4, blue points) using non-linear least squares (trust region method) to obtain estimates of k and Δt.

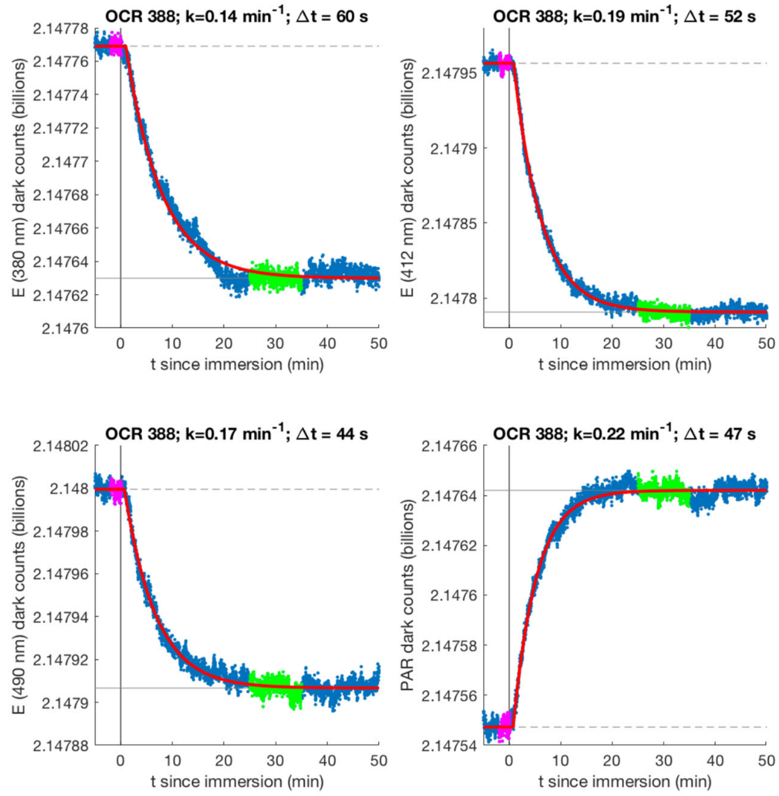

Figure S4. Example fits for calculating heat transfer coefficient k and thermal response time Δt for OCR504 sensor integrated in a RemA housing from timeseries of raw dark counts during laboratory immersion experiments. Data shown are from OCR s/n 388, in RemA s/n 164, later deployed on float WMO 6902670. Each panel shows raw dark counts (blue), with data used to calculate pre-immersion and post-immersion medians in pink and green, respectively. Pre- and post-immersion medians are shown as dashed and solid horizontal black lines.

Using this method, we obtained 52 fits, including replicates, of k and Δt from four channels of 10 RemA sensors with plastic housings (see Table S1). Of these fits, 33 showed strong temperature responses ($|D_0\text{-}D_{eq}|$ > 40000 counts) enabling robust fits. The mean parameters from these fits (±2 standard errors) were k = 0.18 ± 0.01 min^{-1} and Δt = 58 ± 9 s. For the three replicate experiments with the single aluminum RemA sensor, we obtained 9 robust fits and significantly faster mean heat transfer: k = 0.44 ± 0.06 min^{-1} and Δt = 15 ± 2 s.

Section 2. Alternative Procedures to Correct Dark Aging And Temperature Dependence

2.1. Correction of Sensor's Dark Aging Based on Dark Measurements in Day Profiles

In the case of drift measurements acquired for ≤80% of the float lifetime, dark data are extracted from all day profiles following Organelli et al. (2016), which evaluate the normality of the dark distribution by successive Lilliefors tests (α = 0.01, Thode 2002). Then, T_{s_n} for each extracted profile and the associated P_{s_n} are computed following Eqs. (5), (8–9) in the main manuscript. Finally, outliers in radiometry values are removed. Figure S5 shows an example for the float WMO6901647. Please note that if night profiles are available for that given float, those can be included in the analysis.

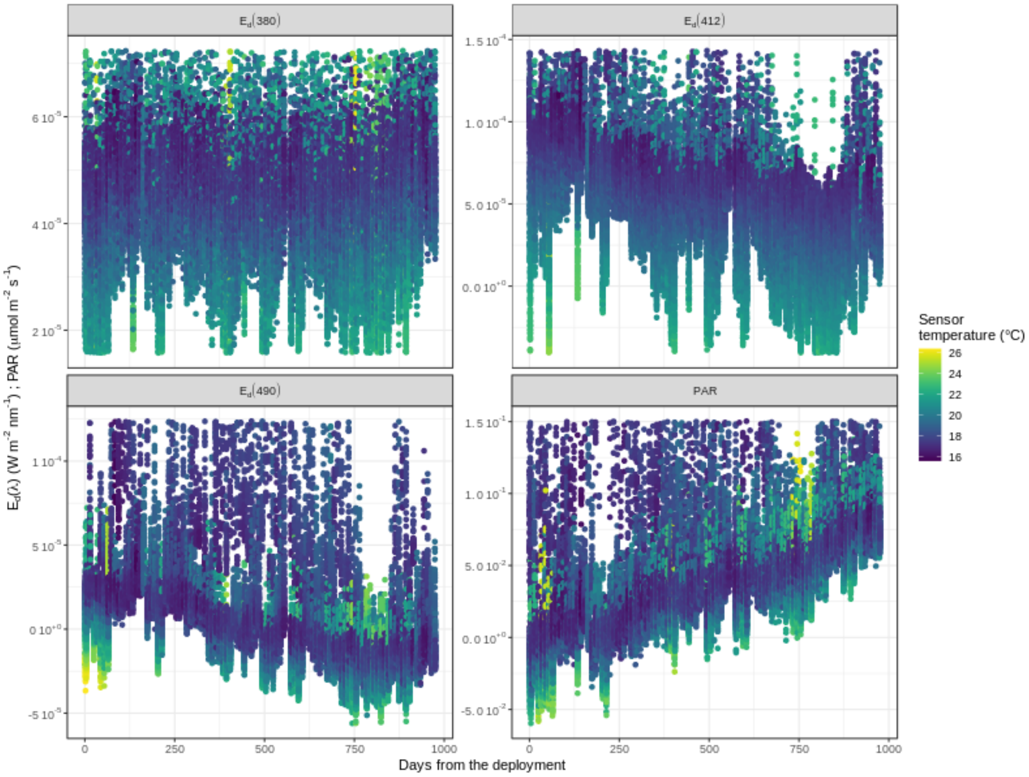

Figure S5. Dark measurements extracted from day profiles acquired by the float WMO6901474 following Organelli et al. (2016).

After the extraction of dark measurements (Figure S5), we follow the same procedure described in Section 3.2.2 of the main manuscript. An example of sensor aging corrected with this method is shown in Figure S6. It is important to note that such a method uses dark data collected up to 250 dbar depth, while main correction protocol described in the main manuscript exploits dark measurements collected at 1000 dbar. The alternative method might thus be more affected by solar irradiance and by a larger range of variability in water temperature, which make it less robust.

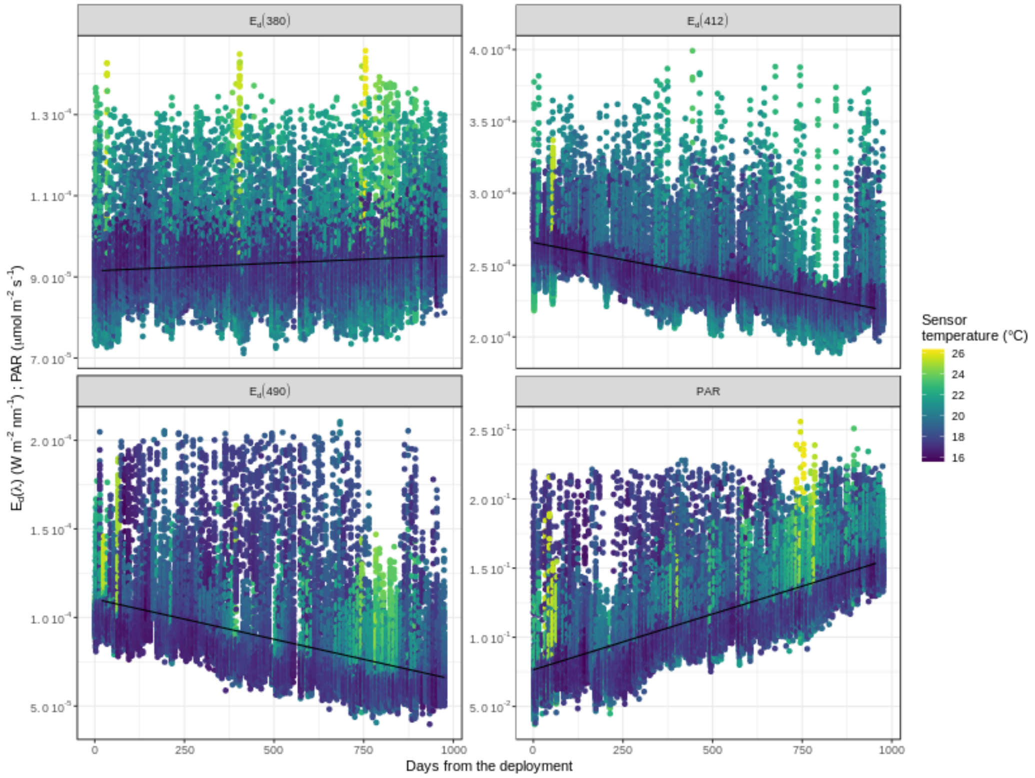

Figure S6. Drift measurements for $E_d(\lambda)$ and PAR as a function of time after estimation at the reference temperature of 5°C. Solid line is the linear fit. Example is shown for the float WMO6901474.

2.2. Correction of Sensor's Dark Temperature Dependence Based on Day Profiles Measurements

This method relies on the assumption that the water temperature dependency of the sensor dark can be highlighted within day profiles by plotting measured irradiance values versus the sensor temperature T_s, because the temperature dependency of the dark should appear as a lower bound of the plotter data.

Aging-corrected dark values within each day profile are extracted following Organelli et al. (2016), i.e., successive Lilliefors tests (α=0.01; Thode 2002) are applied to identify the depth where the dark distribution is close to the normality. The outlier test is not run directly on the obtained data, because this would reduce the range of measured temperature variability. Instead, a median value of all extracted dark measurements within each profile is computed. Profiles with a median value representing an outlier with respect to the median values of all the other profiles of a given float are disregarded. This approach allows us to identify the deepest measured dark signal while excluding the profiles that are strongly affected by light.

Dark data are then sorted by ascending T_s and a moving minimum filter (51-point window) is applied to the radiometry data. Finally, we compute the linear regression of radiometry on T_s *vs.* the filtered data following Equation (20) in the main manuscript. The obtained regression is a linear function vs. T_s of the lower envelope of radiometric data (Figure S7). Knowing that $E_{d_{real}} \geq 0$ and assuming that $[h(T_s,t)]$ is near 1 in Equation (11) of the main manuscript, we have $E_{d_{meas}} - g(t) \geq f(T_s) + \varepsilon$ which means that $f(T_s)$ should

appear as the lower envelope of the corrected signal. Therefore, after the DM operator validates that the regression reasonably represents the lower envelope, we accept the regression as a good approximation for $f(T_s)$. The final correction is applied to all 0-250 dbar profiles following Equation (24) in the main manuscript.

This method provides an estimation of the $f(T_s)$ offset that is slightly lower than the one we could obtain using good night data because of the sensor noise ε. The difference between the two estimates is by definition comparable to the sensor noise and we considered it as acceptable.

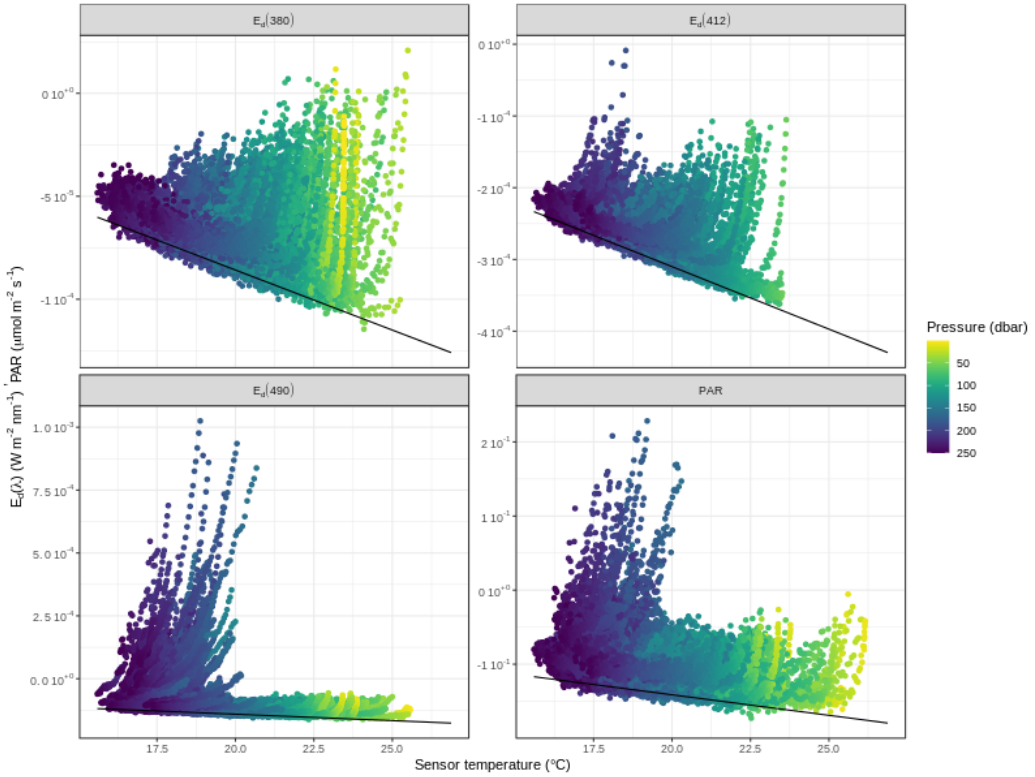

Figure S7. Dark measurements extracted from day profiles acquired by the float WMO6901474 already corrected for the aging of the sensor. Solid black line is the regression to all points on the lower envelope.

Section 3. Example of Failure for Correction Protocols Using Drift and Night Measurements

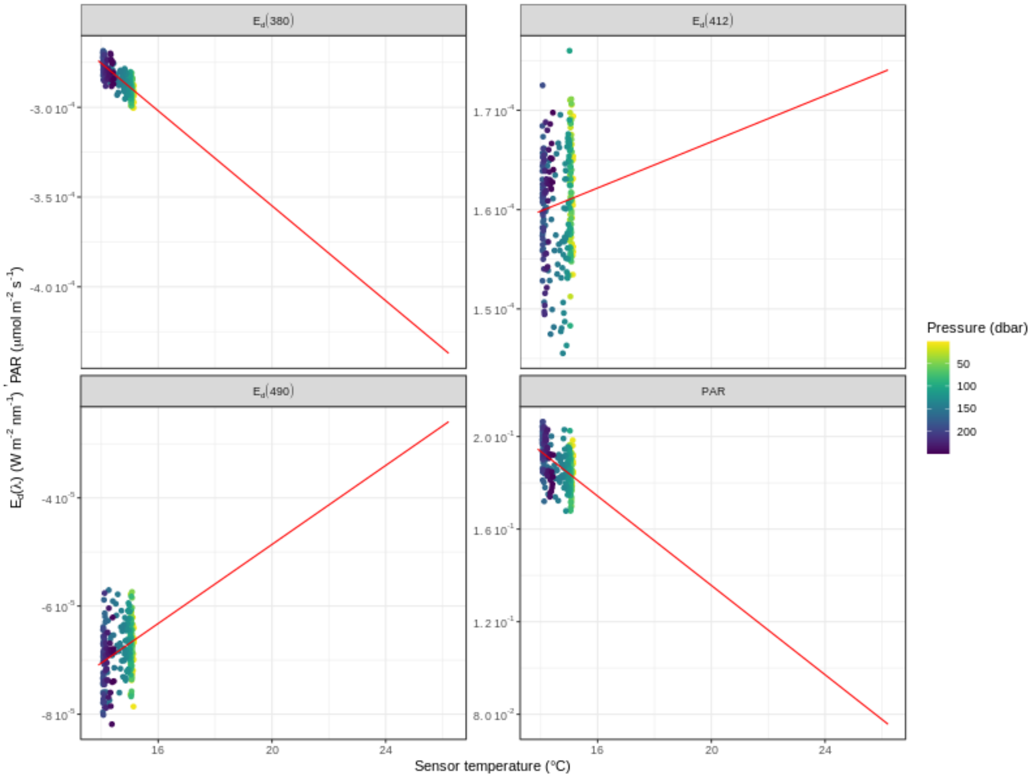

Figure S8. Example of night profiles of $E_d(\lambda)$ and PAR uncorrelated with the sensor internal temperature T_S. Solid red line is the fit to all points extrapolated to cover the full range of temperature encountered by the float. Prior to computing the linear regression, night profiles have been corrected for sensor aging. Example is shown for the float WMO6902903.

Section 4. Magnitude and Variability of the Dark Correction

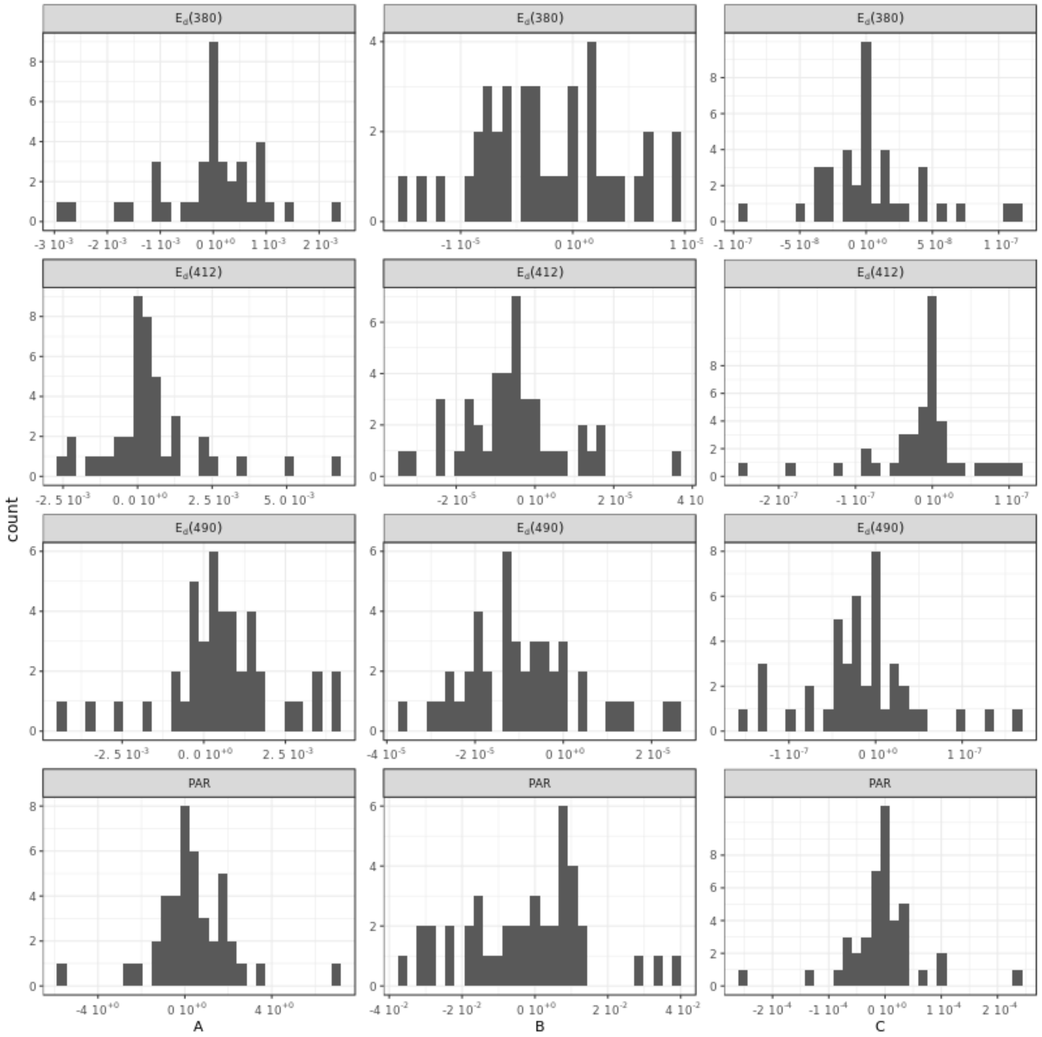

Figure S9. Histograms of A, B, and C coefficients in Equation (24) of the main manuscript used to correct radiometry with drift measurements and night profiles. Cases where a quadratic correction of aging was performed are excluded. A is expressed in radiometry units, B in radiometry units per °C, and C in radiometry units per day. Radiometry units are W m^{-2} nm^{-1} for $E_d(\lambda)$ and µmol photons m^{-2} s^{-1} for PAR. The distributions of the A, B, and C parameters are generally normal. When the C values are equal to 0, the aging correction was abandoned. For a temperature range of 20°C and B = 2×10^{-5} W m^{-2} nm^{-1} $°C^{-1}$ for $E_d(\lambda)$ and B = 4×10^{-2} µmol photons m^{-2} s^{-1} $°C^{-1}$ for PAR, the temperature correction would span 4×10^{-4} W m^{-2} $nm^{-1}$and $8*10^{-1}$ µmol photons m^{-2} s^{-1}, respectively. Over a float lifetime of 4 years and C = 1×10^{-7} W m^{-2} nm^{-1} day^{-1} for $E_d(\lambda)$ and C = 2×10^{-4} µmol photons m^{-2} s^{-1} day^{-1} for PAR, the aging correction would span 1×10^{-4} W m^{-2} $nm^{-1}$and $3*10^{-1}$ µmol photons m^{-2} s^{-1}, respectively.

Section 5. References

Organelli, E.; Claustre, H.; Bricaud, A.; Schmechtig, C.; Poteau, A.; Xing, X.; Prieur, L.; D'Ortenzio, F.; Dall'Olmo, G.; Vellucci, V. A Novel Near-Real-Time Quality-Control Procedure for Radiometric Profiles Measured by Bio-Argo Floats: Protocols and Performances. *Journal of Atmospheric and Oceanic Technology* 2016, *33*, 937–951, doi:10.1175/JTECH-D-15-0193.1.

Thode Jr., H. C. *Testing for Normality. Statistics, Textbooks and Monographs*, 2002, Vol. 164, Marcel Dekker, 479 pp.
